# A Case of Severe and Protracted Headache, Terminating in Tic Douloureux, Successfully Treated

**Published:** 1826-01

**Authors:** A. B. Granville

**Affiliations:** Physician in Ordinary to His Royal Highness the Duke of Clarence, &c. &c.


Art. V.—*A Case of severe and protracted Headache, terminating in Tic Douloureux, successfully treated.* By A. B. Granville, M.D. F.R.S. Physician in Ordinary to His Royal Highness the Duke of Clarence, &c. &c.

It has been stated that tic douloureux, especially of the face, more frequently arises from impaired digestion, than from any local affection, easily detected; and I am inclined to subscribe to a doctrine which seems the offspring of experience, and which daily practice tends to corroborate. But, before the derangement of the digestive functions give origin to such a complaint, it will be observed that many and various other symptomatic ailments of the constitution prevail, equally the effect of that derangement, and the precursors of tic. Not a few of the patients who have suffered from this formidable disorder, and some among whom have perished in consequence of it, might, in all probability, have averted its attacks, had they attended more to the less important disorders under which they laboured for some time before the painful affection of the nerves made its appearance, and directed their whole energy to correct that state of indigestion which gave rise to those disorders. Among these I reckon, as one most deserving of notice, intense and obstinate headache following, or preceding, but invariably accompanying, certain indispositions called *bilious.*

This symptom of deranged action in the stomach, liver, or bowels, (than which there is, perhaps, none of greater importance,) appears, in many instances, to be nothing else than the first stage of *tic douloureux* of some part of the head or face; and I feel warranted in stating further, that a *tic*, or painful affection of the nerves, *external* to the bones of the cranium, will be found, mostly, to follow those acute and intense headaches, arising from indigestion, which, in their progress, have caused affections of some part of the *internal* structure of the head, that have afterwards yielded to an appropriate and vigorous treatment.

My experience in complaints of the stomach during the last ten years, has furnished me with more than one example to illustrate the above practical position; but the object of my present communication being merely to call the attention of the profession to the parental connexion that exists between headache and tic douloureux, and not to enumerate the result of private and public practice, for the sake of numbers,—I deem it proper to confine myself to the narrative of one case only of severe and protracted headache, terminating in the latter disease. It has been my study to give the details of this narrative from written notes taken in the course of this complaint, in order to render it as much as possible practically useful.

A young lady, aged eighteen, moving in the first circles of society, and consequently leading what is called a fashionable life, tall, well made, and free from every (even the most distant) constitutional blemish, either transmitted or acquired; possessing a superior mind, and the most amiable disposition; was placed under my care on the 28th of October, 1824, in consequence of severe headache, which had come on almost immediately after leaving London, at the termination of a very busy season, during which she had largely partaken in the bustling recreations proper for her age. This headache was at first disregarded by the patient, who had seldom or never been heard to complain of illness; but the visible effects of its frequent returns on the expression of her countenance, and other appearances, led to an inquiry, the result of which was a determination on the part of the mother to consult me. I had had the honour of attending, the year before, in conjunction with Sir Henry Halford, a younger sister of the patient, who had also been labouring for some time under a very distressing headache, arising from indigestion; but who, through the care and assiduity of that eminent physician, more than from any feeble effort of mine, had completely recovered. This fact, coupled with another much more important,—namely, the almost sudden death of another daughter a few years before (as I understood), consequent on some affection of the head,—tended naturally to make the parents not less anxious than watchful; and, therefore, no time was lost to obtain medical advice in the present instance.

When I first saw the patient, her countenance was pallid; the skin somewhat tinged with yellow; the pulse natural; the tongue clean; the spirits were good; the nights undisturbed; menstruation went on regularly; and the bowels *were reported* to act properly. There was evidently a degree of languor present, with an indication on the brows of inward suffering, which contrasted singularly with the flow of hilarity that prevailed in her; but I afterwards learned, in the progress of my attendance, to attach a right degree of importance to the latter circumstance, when I found that it was the result of moral fortitude, and not the natural consequence of absence from pain. On being closely examined respecting the nature of the headache from which she suffered, it was stated to consist in sharp shooting pains, occupying, for a short space of time, the left lateral and anterior portion of the head, darting in and away, and occurring at very irregular periods during the day, but never missing any day. Her appetite had, all along, been tolerably good.

Guided by experience in the case of her younger sister, and by what I knew of other instances of a similar kind, I was led to doubt the accuracy of that part of the report which referred to the proper condition of the bowels; and I, therefore, prescribed such medicines as would enable me to ascertain the nature of the evacuations. This I had an opportunity of doing on the following day, when I observed that the fæces were large, figured, spiral, of a dark olive-green, exceedingly compact, shining, and offensive. On inquiry from the female attendant, who narrowly watched the health of the young lady, I learned that such had been the appearances for many months. Who will deny that we had here the key to the whole secret of the headache?

I forthwith regulated her diet, forbade the use of animal food, oftener than three days in the week, and recommended a mixture of pulpy and farinaceous vegetables, dressed in a variety of ways, as a substitute for the other days' fare. The whole quantity of food, also, to be taken in the course of the day was reduced by one-half. An aperient pill without mercury, and a purgative draught, were ordered. At this period of the complaint, there were none of the usual organic symptoms of disturbed action of the liver, such as pain or soreness under the ribs, or between the shoulders; and pressure produced no uneasiness at the pit of the stomach. The third day passed altogether without the slightest headache; the patient expressed herself much pleased with an evident improvement in her feelings, and said she was much better for the diminished quantity of food she had taken. She had, however, had only one motion, and that, too, of the same colour as that of the day before, hard, spiral, and knotted. The purgatives were made stronger; the effects of which were, proportionately, more satisfactory, although the appearance of the secretions was not improved. The headache, however, had not again returned. Being anxious to go back into the country, I allowed her to leave London, on condition of her attending strictly to diet and the use of purgative medicines.

On the 20th of November, she again came up to town, to report progress. Her countenance was astonishingly improved. She was greatly pleased with the regularity of her diet, and the pleasant feelings consequent upon it. Her bowels had acted regularly twice, and even three times every day; and she had not experienced the slightest degree of headache. She had once been at a ball in the country, where she had danced a great deal, without experiencing any inconvenience. I substituted a milder purgative for the stronger pills, and recommended perseverance in the reduced diet.

On the following week, I found her countenance very yellow, the bowels sluggish, her pulse quick; but free from headache, and still in high spirits. One circumstance, however, attracted my attention, and that was the exquisite soreness of that portion of the scalp which covers the right fronto-parietal region, so that she could not bear to touch it, and felt as if the hair was pulled tightly backwards. I changed the purgative medicines, which gave rise to an immediate improvement, from a more brisk action on the intestines being produced by them.

Another fortnight elapsed without any headache; the soreness of the scalp only continuing. The patient again indulged in her favourite amusement, dancing, without any headache ensuing.

About the middle of the first week in December,—the complexion having in the mean while improved, the bowels acting pretty regularly, and the head continuing free from pain,—the purgative medicines were gradually discontinued. During the whole of this period, the greatest attention was paid to diet and regimen. In a few days, however, the bowels again became costive: one evacuation only occurred in the day; and this change in the secretions was followed by a return of shooting pains in the head, and an increase of soreness in the scalp, with a discoloration of the countenance. She, therefore, returned to the use of stronger purgatives, which soon brought an alleviation of the symptoms. The secretions appeared very unfavourable, and plainly denoted considerable derangement of the digestive organs. The patient was, at this time, observed to be restless and agitated in her sleep; and the headache invariably assailed her on waking, though it would, occasionally, disappear in the course of the morning, again to return once or twice, for two or three hours at a time. Satisfied, by a fresh inquiry into the symptoms, that we had only a case of *sympathetic* cephalalgia to deal with at present, I contented myself with persevering in my original views and treatment, which failed not to produce, in due time, a proportionate gradual amendment; so that, under the date of the 23d of December, I find in my note-book the following memorandum:—" Miss ——— looks much better. She has had occasional shooting pains in the head, which have got well by riding on horseback, or disappeared shortly on taking a nervous draught. The pain on these occasions is *said to be different* from that under which she laboured before. The state of the bowels is greatly improved; they act freely, and twice a-day. Pulse full and quick; she menstruates regularly."

About this period she left town; and I did not again see her until the 9th of January, when I found her labouring under much more alarming symptoms than I had seen her to suffer from before; symptoms which seemed to be the precursors of a serious and complicated disorder, as will be seen from the sequel of the present narrative.

It appeared, on inquiry, that, being free from all pain, and feeling quite well, our patient had somewhat relaxed in her attention to diet and rules for exercise, and had indulged in the favourite amusement of waltzing, at a ball given at Bushy Palace. The immediate effect of this was giddiness, and sickness at the stomach on the succeeding days, which induced her relations to bring her to town. On my visiting her, I found her labouring under excruciating headache; and vomiting, with violent efforts, large quantities of bile. The tongue was foul; the bowels costive; pulse soft, and eighty-four. Having, in the course of my attendance, ascertained beyond a doubt, on more than one occasion, that calomel in particular, and even the blue pill, however indicated by the present symptoms, acted on her constitution most injuriously, by producing febrile excitement, dejection of spirits, acute pain at the pit of the stomach, and an increase of headache, I was debarred from the assistance of those powerful auxiliaries in combatting what I considered a severe bilious attack: I therefore confined myself to the employment of extract of colocynth, rhubarb, and neutral salts. These, however, produced so trifling a relief, that, on the following day, I was driven to a fresh, though cautious, trial of the last-mentioned mercurial preparation; from which some considerable good effects were obtained, as far as relates to the condition of the bowels, yet not without great disturbance of the nervous system. The secretions were highly offensive, and of a bad character, at first, but they improved somewhat in a few days; and, with that improvement, the headache diminished.

A short time after this, a change took place in the manner of the attack. After a restless night, during the first part of which the pain in the head would gradually subside, perspiration came on towards morning, and sleep followed, which continued till nine or ten o'clock. She would then awake, free from headache, and remain so until she began to exert herself in getting up and dressing; when the pain in the head suddenly came on, preceded by chilliness, and attended with a sensation of weight. This continued, and indeed kept increasing, until dinner-time, when it generally left the patient for an hour or two, again to reappear for the evening. While the pain in the head subsisted, every noise, and even strong light, affected the patient, and made her worse. The coming on and going off of the pain was invariably sudden, but there was no beating in the affected part. The pulse was, on all these occasions, deep, slow, somewhat full, and irregular, and with a rhythm which musicians call *stentato*. The soreness of the scalp over that part of the head which was affected with pain, was not so great as when it had existed without the headache. The bowels, notwithstanding the purgative system and attention to diet, had again got into an unsatisfactory state. The evacuations were in the highest degree improper and offensive; and, on the morning of the 14th of January, bilious vomitings came on again ; so that, had there been any febrile indication, with all the other symptoms present, one would have taken this to be either a continued or a bilious remittent fever.

About this time, the obstinacy of the complaint, and particularly of that one symptom, headache, which had accompanied the fatal disorder in the patient's sister a few years before, began to alarm her parents. The assurance given them that the affection in the head was dependent on that of the stomach, began to fail in tranquillising their fears, inasmuch as the same assurance had precisely been given in the case of the daughter they had lost, up to the very last hour of its formidable career. It was evident, moreover, that, although symptomatic, the complaint in the head threatened to assume a character of local derangement; and that fulness was obtaining in the vessels, which mere purging and diet appeared not wholly to prevent. Bleeding from the arm was therefore proposed, and carried into effect. A very sensible general practitioner, Mr. Sheldon, who had been for years in attendance on the family, bled the patient on the evening of the 14th, in my presence; when the blood flowed freely, but thick and black, and of a remarkably high temperature. The pulse rose immediately after the operation, and the pain in the head was much diminished before morning.

At the end of the second day after the operation, the headache had wholly disappeared. The bowels acted under less powerful medicines, which circumstance induced me to suspend the use of the bluepill, and to recommend, occasionally, a nervous draught.

This amendment subsisted, without any remarkable deviation, for seven days; during which period I observed that her pulse continued much fuller than it was previous to the bleeding, yet not more frequent; that, whenever she took a stronger dose of medicine, pain in the head came on after the third and fourth motion, for an hour or two; and, lastly, that she now and then would complain of chilliness, particularly in the back and along the spine, from the occiput down to the loins. During the whole of this period, the nights were particularly good, the complexion of the patient improved, and there did not appear any visible diminution of either flesh or strength. The spirits continued excellent.

Notwithstanding all this improvement and apparent recovery, I could not be prevailed upon to relax in my orders as to the diet of the patient; but, some indulgence in that respect having been allowed, from a mistaken direction, a repetition of all the worst symptoms took place almost abruptly. I had left the patient on the 20th in the very best state; and, on my visit the following day, I found her labouring under all the symptoms which had first appeared on the 9th instant; such as sickness, headache, quick pulse, pain at the pit of the stomach, foul tongue, thirst, languor, great depression of spirits, with (now for the first time) exquisite soreness throughout the region of the liver. It was, indeed, remarkable that the latter symptoms had never been present before, although I had directed my attention to, and felt the part frequently, for the purpose of ascertaining if any mischief existed there.

This case had now become so discouraging, from the length of time it had lasted, and the little impression the medicines had made on the disease: and I felt so much the high responsibility of dealing with it single-handed, where the eyes of many anxious relatives and connexions were fixed upon me, all dreading the worse consequences, that I forthwith proposed to the mother a consultation; when she selected Dr. Maton for that purpose. In the mean while, following up what I considered a just indication, and once more declaring my opinion to be that the disease would ultimately prove to be wholly dependent on the defective condition of the digestive organs, I administered an emetic of twenty grains of ipecacuanha, which cleared the stomach effectually, and at the same time acted on the bowels. Were the quantity of bilious secretion thrown out on this occasion to be stated, it might appear incredible. Towards the evening of the same day, every unpleasant symptom had subsided; and, on the following day, the patient was so much better, that the parents spontaneously proposed a postponement of the consultation. About this time menstruation came on, and flowed regularly, although its proper period had elapsed by fifteen days. Towards the conclusion of this operation, headache occurred again, followed by vomiting. The pulse was now ninety-two: I had never felt it so high before. There were alternate paroxysms of heat and cold. No motion from the bowels, and the urine highly coloured.

On the 24th, Dr. Maton was kind enough to meet me in consultation. He inquired minutely into the history and every particular circumstance of the case, and agreed with me that to the state of the stomach we were to look for an explanation of that complicated series of symptoms which made the young lady's malady peculiarly interesting. It was agreed to have again recourse to effectual purgation, and a rigid diet. A pill of rhubarb and extract of hyoscyamus, which had generally agreed, and produced comfortable sleep at night, was continued; and, in addition, a draught was ordered, consisting of aquæ anethi f.℥jss.; magnes. sulph. ʒij.; and magnes. carb. gr. x. bis in die. No meat or fish was allowed. On the following day, after a copious evacuation of fœtid and bilious fæces, considerable amelioration of all the symptoms took place. I persevered in this plan, with the same good effects, for three days after the consultation; increasing, rather, the strength of the aperient pills, and forbidding all but liquid food.

On the 29th, the following memorandum appears in my notebook:—"Miss —— has had a relapse. Awoke at four o'clock A.M. with sickness at the stomach, and a bitter taste in the mouth. Had four bilious evacuations. The urine is highly turbid and sedimentous; pulse slow, rather full. Complexion deeply yellow. Much headache, and shooting pains from the forehead to the occiput. The purgatives to be continued; no food whatever to be allowed. In the evening, she was again much better, and in high spirits; slept well the succeeding night, and awoke next morning refreshed, and with very little headache."

This amendment continued until the 3d of February, when, for the first time, she experienced a pain in the left side of the face, neck, and the external parts of the eye, which, being attributed to a draught of air, was considered as rheumatic; and bags of hot chamomile flowers were ordered by Dr. Maton to be applied to it.

On the 4th, I found the patient with the rheumatic pain much increased, although the application recommended by Dr. Maton had, in the first instance, produced alleviation. The whole of the left side of the body, from the temple downwards, was highly painful and sore to the touch. There came away from the stomach a quantity of clear water, having a saltish taste. The bowels were regular, but the pulse irregular and slow; no headache. I prescribed as follows:—Pilulæ saponis cum opio, gr. v.; pulveris Jacobi veri, gr. iij. statim; and Mæ. camph. f.℥j. aquæ ammon. acetatis, f.ʒss. Haustus horâ, pot pilulas, sumendus et quintis horis repetendus. The patient was desired to remain in bed, with a view to encourage perspiration. In the evening, I found her free from pain, (having slept several hours,) and in a profuse perspiration. The sickness had ceased, but considerable swelling of the parts before affected had supervened.

I have been thus minute in detailing the course of this singular complaint, in order to show the slow gradations by which it passed from costiveness to indigestion,—from indigestion to headache,—from headache to rheumatic affection of the muscles of the head, face, and neck,—and from this to a more intense pain in the head, accompanied by weight, tintinitus aurium, deafness, shootings in the left eye, with a constant flow of tears from it, a full pulse, impossibility of laying the head on the pillow, or to leave the bed, and total inability to bear light; all which latter aggregate symptoms had made their appearance insidiously, creeping on slowly, on the subsiding of the rheumatic pain in the face, at the time of the last report.

The case was now reduced to its most distressing limits; and, certainly, no cautious practitioner would have presumed to decide, under such accumulated circumstances, that the still subsisting symptoms of bilious derangement were the positive cause of the increased pain in the head, rather than the effect of it. I determined, therefore, to be on my guard against both; and, in one of the intervals which elapsed between the consultations, when symptoms of an alarming nature occurred with regard to the head, which could not be accounted for by any irregularity of the stomach or bowels, I directed blood to be taken from the left temple and from behind the left ear, by cupping-glasses. This operation I had occasion to direct Mr. Mappleson to perform twice, at different times, to the extent of twelve ounces; and at each time immediate relief followed. The consulting physician approved of this course, and, in conjunction with myself, ordered, on another occasion, when severe sincipital and temporal headache, accompanied with heaviness and soreness of the scalp prevailed, to apply twenty leeches; and, a few days afterwards, a seton to be passed through the nape of the neck. The neutral salts, particularly the sulphate of potass, were continued. The diet at this period was watery. By this plan of treatment we felt confident we had saved the patient from much serious mischief.

In this manner we proceeded for some time, when the complaint, again changing character, appeared to quit the head, and direct, once more, its whole strength to the stomach and to the region of the liver, which latter became highly painful. Having tried for the twentieth time, with no less injurious effects to the nerves, the pilula hydrargyri, and even the oxymuriate of mercury, we resolved on rubbing in the mercurial ointment on the region of the liver. This was done for ten or twelve days very freely, but no sensible effect was produced on the mucous membranes. The constitution seemed unsusceptible of the ordinary action of mercury; while, on the other hand, it became greatly disturbed,—alarmingly so, indeed, whichever preparation of that medicine was employed. One advantage, however, we derived from this course,—namely, the removal of the enlargement and pain in the region of the liver. But, on these symptoms disappearing, the headache again returned, and with greater violence than ever; the stomach and bowels becoming once more deranged at the same time, and to a greater degree than before. We now suspended the external use of mercury altogether, and carried off by other means prodigious quantities of black and green bile by stools; three or four of which were obtained every day.

To go on detailing these alternated attacks, and remissions of headache and disorder in the stomach, through the whole period of their occurrence, which lasted until the middle of April, would seem tedious, although the details might be deemed interesting to a practitioner placed in a similar situation with ourselves; when any encouragement to be derived from the knowledge of a similar case, its treatment, and successful termination, would have been hailed by us with satisfaction. Our situation, indeed, was one of the greatest anxiety, and we could not behold the slow sinking of our patient, her emaciation, constant sufferings, total depression of strength and spirits, and an almost uninterrupted febrile condition of the constitution, lasting for upwards of six months, to which was now superadded an unpleasant cough, without feelings of alarm. We had succeeded, indeed, in tracing the connexion of the cephalic symptoms with those affecting the digestion;—we had effectually checked, by active and prompt measures, that period of danger arising from a temporary fulness of the vessels of the head, which had, at one time, placed the life of the patient in jeopardy;—we had ascertained that the head-aches were under the control of purgative and alterative medicines; although we found out, at the same time, that what should have been our most powerful auxiliaries in this class, the mercurial preparations, acted mischievously on the system, and were consequently inadmissible. But we had, on the other hand, before us an extenuated patient, with a constitution broken down by unwearied sufferings, watchfulness, low diet, and fever; and still the call for purgatives and other lowering medicines continued imperative, for bad secretions still flowed, and the headache ceased not.

It was at this conjuncture that, watching by her side one day, when the patient laboured under what she called an agonising pain in the head, I endeavoured to procure from her a clearer account of her sufferings than she had lately been able to give us. She began by stating that the pain in the head, for the last five weeks, had been very different from that which she had experienced during the first five months of her complaint; that it was now, as it were, under her hair,—arose in the back of her neck,—travelled over the head, confining its course chiefly to the left side of it,—descended over the eyebrow, passed through the eye, which became painful to a degree, and shed tears,—penetrated the upper jaw,—made all the teeth of that side highly painful,—and, lastly, reached the long muscles of the neck. Thus spread, and occasionally involving the left ear, the pain would last for half an hour, and then gradually disappear, leaving a sensation of coldness and a tired feel in the parts, During the paroxysm, the whole extent of the affected side was so exquisitely tender, that the slightest touch could not be borne,—the eye could not be opened,—the face could not be laid on the pillow, and the slightest noise would increase her sufferings. In the course of this clear narrative, two exacerbations, and one intermission or remission of pain, occurred. I felt the pulse then: it appeared not to be affected in any way by what was taking place in the head. As these were the symptoms that had prevailed for the last five weeks, more or less, it struck me that the disease might now be considered as reduced to its simplest elements, and as having become a *tic douloureux*. I hastened, therefore, to communicate my observations to Dr. Maton at the next consultation, and proposed to try the use of the carbonate of iron, in scruple doses three times a-day, a rhubarb pill at night, and an enema to open the bowels in the morning.

This plan having been acceded to, and carried into execution, we were not long in witnessing its good effects. There was a cessation of the pain in a few hours, and in two days more the patient was able to leave her bed with assistance, and walk, for the first time, out of her bed-room, bearing a strong light without much discomfort. After a few days more, the dose of the carbonate of iron was increased to half-a-drachm three times a-day, with a corresponding increase of benefit; so that three or four days would elapse without any pain. The bowels were all along kept freely open by rhubarb only, and the enema. Three, and frequently four, motions were thus obtained daily; which were, of course, deeply tinged by the iron, and very offensive. The medicine never appeared to raise the pulse, although it was in due course increased to one drachm three times a-day; from which time until the 14th of May, 1825, (a period of three weeks,) that quantity of carbonate of iron was continued as before directed, and no attack of tic occurred except once, when the bowels had not acted for two days.

The period of convalescence, after the cessation of the spasmodic pain in the head and face, was short, and the recovery so rapid as to astonish every one. The patient, as soon as she was able, was sent out of town; and is now, and has been for some months, not only free from all disease, with her digestive powers perfectly restored, but better, even, than she had been for many years before.

The length to which this narrative has extended, precludes the possibility of any remarks on the singular events it embraces, were I even inclined to indulge in them. I have been taught by them more than one lesson in practice, by which I hope to profit; and I trust that the perusal of it may prove equally instructive to others. It is gratifying for the medical attendants in this case to think that, in the management of its various difficulties, they have found practical applications,—a dispassionate examination of symptoms, unbiassed by theories or systems, —a cordial co-operation,—and the benefit of previous experience, to be by far the best resources on which to rely for the discharge of their duties towards their patient.

*Grafton street; 10th December,* 1825.

